# Expression of platelet derived growth factor family members and the potential role of imatinib mesylate for cervical cancer

**DOI:** 10.1186/1475-2867-6-22

**Published:** 2006-10-02

**Authors:** Lucia Taja-Chayeb, Alma Chavez-Blanco, Jorge Martínez-Tlahuel, Aurora González-Fierro, Myrna Candelaria, Jose Chanona-Vilchis, Elizabeth Robles, Alfonso Dueñas-Gonzalez

**Affiliations:** 1 Unidad de Investigación Biomédica en Cáncer, Instituto Nacional de Cancerología/Instituto de Investigaciones Biomédicas, UNAM, Mexico; 2 Division of Clinical Research, Instituto Nacional de Cancerología, Mexico; 3 Department of Pathology, Instituto Nacional de Cancerología, Mexico

## Abstract

**Background:**

Despite significant achievements in the treatment of cervical cancer, it is still a deadly disease; hence newer therapeutical modalities are needed. Preliminary investigations suggest that platelet-derived growth factor (PDGF) might have a role in the development of cervical cancer, therefore it is important to determine whether this growth factor pathway is functional and its targeting with imatinib mesylate leads to growth inhibition of cervical cancer cells.

**Results:**

PDGF receptors (PDGFR) and their ligands are frequently expressed in cervical cancer and the majority exhibited a combination of family members co-expression. A number of intronic and exonic variations but no known mutations in the coding sequence of the *PDGFRα *gene were found in cancer cell lines and primary tumors. Growth assays demonstrated that PDGFBB induces growth stimulation that can be blocked by imatinib and that this tyrosine kinase inhibitor on its own inhibits cell growth. These effects were associated with the phosphorylation status of the receptor.

**Conclusion:**

The PDGFR system may have a role in the pathogenesis of cervical cancer as their members are frequently expressed in this tumor and cervical cancer lines are growth inhibited by the PDGFR antagonist imatinib.

## Background

Cervical cancer remains as one of the biggest killers of women worldwide [1]. The oncogenic role of human papillomavirus for the development of this cancer has been vastly demonstrated [2], however, an unknown number of genetic [3] and epigenetic [4] defects are also needed for tumor formation, such as alterations in the epidermal growth factor receptor (EGFR) and other membrane receptors [5]. Abnormalities in platelet derived growth factor (PDGF) family members have been underscored as important players for neoplasms such as meningiomas, gliomas, melanomas, neuroendocrine tumors, sarcomas and ovarian, pancreas, gastric, lung, breast and prostate carcinomas [6-8]. PDGF is a potent mitogen and chemotactic factor for mesenchymally derived cells, and was one of the first polypeptidic growth factors identified that signals through a cell surface tyrosine kinase receptor (PDGFR), to stimulate various cellular functions including growth, proliferation, and differentiation. Since then, several related genes have been identified constituting a family of ligands (PDGFA B, C and D) and their cognate receptors (PDGFR alpha and beta) [9-11]. PDGF isoforms are dimeric molecules that bind two receptors simultaneously which dimerize upon binding. These receptors may generate homo- or heterodimers resulting in the next combinations PDGFRαα, αβ or ββ. The PDGFRα is able to bind to PDGF chains A, B and C, while PDGFRβ binds only PDGF chains B and D [12]. Interestingly, the heterodimeric receptor complex αβ have different properties compared to the homodimers [13].

The recent availability of potent and specific tyrosin kinases inhibitors such as imatinib mesylate, which was developed as an ATP competitive inhibitor of ABL tyrosine [14] that, at concentrations required for inhibition of Bcr-Abl, also inhibits PDGFR and c-Kit [15], has revived the interest on the PDGFR as potential therapeutic target for several neoplasms, including solid tumors [16,17]. Regarding cervical cancer, the role of the PDGFR system remains to be explored. So far there are some indications that PDGFR could participate in the HPV-driven carcinogenic process. It has been shown that transformation by the bovine papillomavirus type 1 (BPV-1) E5 protein binds to and activates PDGFRβ inducing its dimerization and sustained activation similarly to PDGF [18,19]. Further, it was proven that the E5-induced tyrosine phosphorylation or mitogenic activity of the receptor is ligand PDGFR independent. Thus, it seems that PDGFRβ is the main cellular target of the BPV-E5 oncoprotein [20,21]. Little is known regarding the PDGF and PDGFR expression in cervical cancer. There is only one small study where the expression of the PDGFR protein was investigated in 11 patients with mild, moderate or severe dysplasia as well as four invasive cervical cancers. Expression of PDGFRβ was observed at high levels in low-grade cervical lesions, while expression was depressed in high-grade lesions, suggesting a role for PDGFR during early stages of cervical carcinogenesis [22].

On the other hand, PDGFRβ participates in the regulation of interstitial fluid pressure (IFP), by modulating the tension between cells and extracellular matrix structures [23]. Subsequently it was demonstrated that IFP is elevated in most malignant tumors, mainly as a result of the abnormal tumor vasculature that develops from unregulated angiogenesis, resulting in a diminished hydrostatic gradient from capillary to interstitium and thereby impairing the exchange of solutes over the capillary membrane, creating a physiological barrier to tumor uptake of drugs from circulation [24,25]. Increased interstitial fluid pressure has also been demonstrated in cervical cancer patients [26]. Interestingly, a prospective study in 102 cervical cancer patients, demonstrated a strong independent prognostic value of pre-treatment IFP measurements in tumors. Patients with high IFP are significantly more likely than those with low IFP to recur after radiotherapy and die of progressive disease [27].

It has been demonstrated that not only overexpression of PDGFR participates in carcinogenesis; also genetic changes have a role in this process. So far, Heinrich demonstrates that activating gene mutations of *PDGFRα *in GISTs have biological consequences, highlighting a crucial role for PDGFRα in the pathogenesis of a solid tumor [28]; in fact some of this mutations induce imatinib resistance [29].

All together these data clearly suggest that blocking the PDGFR system would be of terapeutical value in cervical cancer, hence in this work we analyzed the expression of PDGF family members as well as the mutational status of the *PDGFRα *gene in a series of cervical cancer cell lines and primary tumors of cervical cancer patients. In addition, the growth inhibitory effect of imatinib was also investigated in a cervical cancer cell line.

## Results

### Expression of PDGF family members in cervical cancer cell lines

Expression of PDGF ligands and receptors was investigated in eight cervical cancer cell lines. The results, presented in Table 1 demonstrate that all cell lines tested, expressed PDGFRα but none expressed PDGFRβ. In regard to the ligands, the PDGFB was present in five (62.5%) out of eight cell lines whereas 50% (4 out of 8) of the cell lines expressed PDGFA. Interestingly, all cell lines co-expressed the PDGFRα with at least one of the ligands. There was only one cell line (CaLo) which co-expressed both ligands along with the receptor. Hence this cell line was chosen for further testing.

**Table 1:** Expression of PDGF family members in cervical cancer cell lines.

Cell line	PDGFRα	PDGFA	PDGFRβ	PDGFB
C33A	+	-	-	+
INBI	+	-	-	+
ViPa	+	-	-	+
ViBo	+	-	-	+
HeLa	+	+	-	-
SiHa	+	+	-	-
CaLo	+	+	-	+
CasKi	+	+	-	-

Cell lines were analyzed by immunocytochemistry. CaLo cells co-expressed the receptor alpha and both ligands. Positivity was considered when 5% or more of the cell population stained.

### Expression of PDGF family members in primary tumors

**Figure 1:**
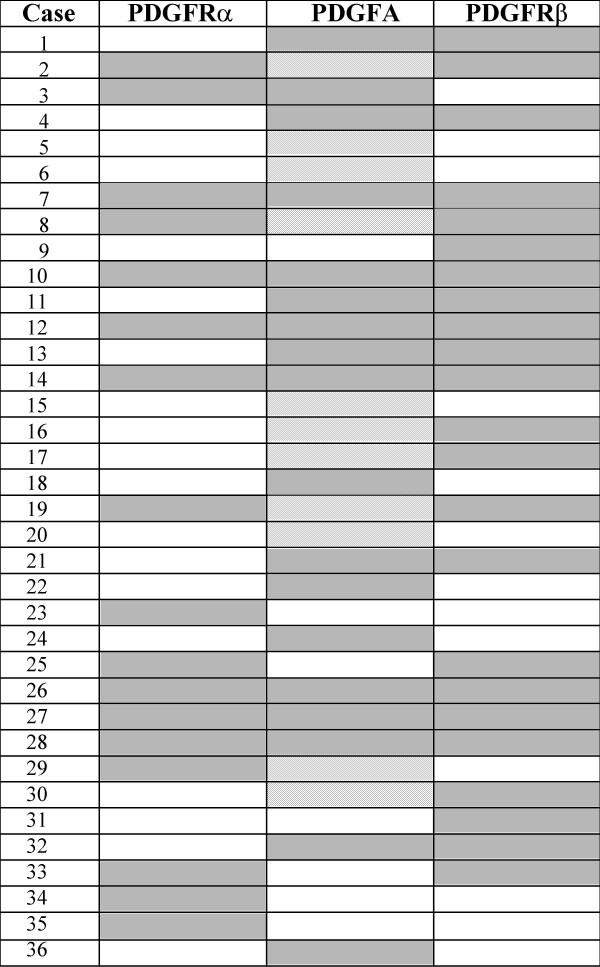
Expression of PDGF family members in primary tumors. In this figure we show the co-expression of receptors and ligand in each sample. Gray boxes indicate positivity, white negativity and line-drawing not done. Summary of the results is: **PDGFRα**: 15 cases (41.6%) were positive in tumor cells, of these, 3 cases show expression in tumor and stroma cells. Two cases were positive only in stroma (5.5%). **PDGFRβ**: 19 cases (52.7%) were positive in tumor cells, of these 13 samples show expression in both tumor and stoma cells. Four tumors were positive only in stroma cells (11.1%). **PDGFA**: 15 cases (60%) were positive at the tumor component, 14 of them in both tumor and stroma; 3 cases (12%) positive only in stroma. **PDGFB**: All negative

**Figure 2:**
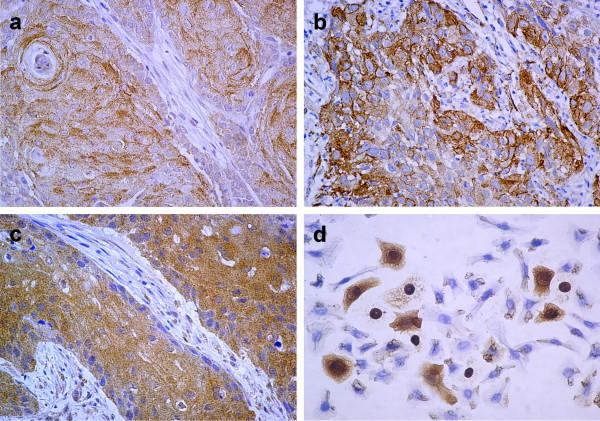
Representative samples of primary tumors expressing: a) PDGFRα, b) PDGFA and c) PDGFRβ. (100X). d) shows the staining for PDGFRα in the cell line CaLo (400X).

The immunohistochemical expression of PDFG and PDGFR family members was evaluated in the primary tumors of 36 patients. Biopsies were taken before the patients received any anticancer therapy; mean age for the patients was 40.8 years; 6 patients were staged as IB2-IIA, 14 as IIB and 16 as IIIB; 32 and 4 were histologically classified as squamous and adeno/adenosquamous respectively. The immunohistochemistry results are shown in Figure 1. Overall, PDGFRα was expressed in the malignant cell component of the sample in 15 (41.6%) of cases, 3 of them (8.3%) showed positive reaction in tumor cells as well as in stroma; and in 2 cases (5.5%) only in the stroma Regarding the PDGFRβ, 19 samples (52.7%) expressed the receptor in the tumor component, 13 out of the 19 samples showed signal in tumor and stroma; 4 cases (11.1%) were positive only in the stroma. PDGF ligands were evaluated only in 25 samples because of sample limitations. PDGFA was expressed in 15 cases (60%) in the tumor cell component; 17 samples (68%) stained in the stroma, 14 of them were positive in both components, while 3 (12%) only in the stroma. None of the samples expressed the PDGFB. Representative cases are shown in Figure 2.

### Mutational analysis of the *PDGFRα *gene

To further characterize the potential participation of PDGFR in cervical cancer development and to determine the presence of gene-activating mutations or mutations associated to imatinib resistance, we performed a mutational analysis of the *PDGFRα *exons 12 and 18 in the DNA extracted from the 8 cell lines and the frozen cervical tumors. Table 2 shows that cell lines ViPa, HeLa, CaLo and INBL were wild type for the exon 12 and presented a silent mutation in codon 824 C>T leading to a Val>Val change (SNP rs10015469, previously reported) in exon 18. On the other hand, C33, CasKi and SiHa cells were wild type for exon 18 but showed a G>A in codon 571 leading to a Glu>Lys substitution (unreported changes). Interestingly ViBo cell line had three silent mutations at exon 12 and two intronic variations, whereas in exon 18 no exonic variations but two intronic variations were found. Regarding the analysis of the 17 tumor samples, all were wild type for exon 12. At exon 18, seven patients were wild type and ten had the same silent mutation found in cell lines at codon 824 (Table 3). Regarding normal tissues, we performed the sequence analysis in three cervical tissues and in the lymphocytes from 8 healthy donors, the three cervical samples had the codon 824 silent mutation in exon 18 and, two of them presented an intronic polymorphism in exon 12 (Table 3). All the lymphocyte donors were wild type for exon 12 and three of them had the silent mutation at codon 824 (data not shown).

**Table 2:** Sequence analysis of PDGFRα exons 12 and 18 from eight primary cervical cancer cell lines.

**CELL LINE**	**EXON 12**				**EXON 18**			
	**localization**	**change**	**position**	**amino acid**	**localization**	**change**	**position**	**amino acid**

ViBo	Exon	G>A CCA >CCG	Codon 567	Pro > Pro	Intron	C >G	IVS18 +21	---
	Exon	G>A CCA >CCG	Codon 577	Pro > Pro	Intron	A >G	IVS18 +25	---
	Exon	A>G TCA >TCG	Codon 584	Ser > Ser	-----			
	Intron	A> G	IVS12 +17	----	-----			
	Intron	T> C	IVS12 +35	----	-----			
ViPa	WT				Exon	C >T GTC>GTT	Codon 824	Val > Val
HeLa	WT				Exon	C >T GTC>GTT	Codon 824	Val > Val
CaLo	WT				Exon	C >T GTC>GTT	Codon 824	Val > Val
INBL	WT				Exon	C >T GTC>GTT	Codon 824	Val > Val
C33	Exon	G >A GAA>AAA	Codon 571	Glu > Lys	WT			
Caski	Exon	G >A GAA>AAA	Codon 571	Glu > Lys	WT			
SiHa	Exon	G >A GAA>AAA	Codon 571	Glu > Lys	WT			

WT: wild type; IVS: intronic variation sequence. We found 7 intronic and 5 exonic variations sequences [RefSeq accession D50017]. None of the intronic variations have been reported: for exon 12 we found, A >G IVS12+17 and T >C IVS12+35, and for exon 18, C >G IVS18+21 and A >G IVS18+25. The exonic changes were: G >A in codon 567 CCA >CCG, Pro>Pro [rs1873778] synonimous; G>A codon 577 CCA >CCG, Pro >Pro (unknown) synonimous; C >T in codon 824 GTC >GTT, Val >Val [rs10015469] synonimous; G >A codon 571 CCA >CCG, Glu > Lys (unknown) not deleterious; and A >G in codon 584 CCA >CCG, Ser > Ser (unknown) synonimous.

**Table 3:** Sequence analysis of PDGFRA exons 12 and 18 from primary cervical cancer tumors and normal cervix samples.

**SAMPLE**	**EXON 12**				**EXON 18**			
	
	**localization**	**change**	**position**	**amino acid**	**localization**	**change**	**position**	**amino acid**
CU-194	WT				WT			
CU-493	WT				Exon	C >T GTC>GTT ht	Codon824	Val > Val
CU-693	WT				WT			
CU-794	WT				Exon	C >T GTC>GTT hm	Codon 824	Val > Val
CU-1094	WT				Exon	C >T GTC>GTT ht	Codon 824	Val > Val
CU-1194	WT				Exon	C >T GTC>GTT ht	Codon 824	Val > Val
CU-1494	WT				WT			
CU-1593	WT				Exon	C >T GTC>GTT ht	Codon 824	Val > Val
CU-2294	WT				Exon	C >T GTC>GTT ht	Codon 824	Val > Val
CU-3194	WT				Exon	C >T GTC>GTT hm	Codon 824	Val > Val
CU-3293	WT				Exon	C >T GTC>GTT ht	Codon 824	Val > Val
CU-3994	WT				Exon	C >T GTC>GTT hm	Codon 824	Val > Val
CU-4594	WT				WT			
CU-4793	WT				WT			
CU-4994	WT				WT			
CU-5393	WT				WT			
CU-5593	WT				Exon	C >T GTC>GTT ht	Codon 824	Val > Val
CN-893	Intron	C > A	IVS12 +22	------	Exon	C >T GTC>GTT ht	Codon 824	Val > Val
CN-993	Intron	C > A	IVS12 +22	-----	Exon	C >T GTC>GTT ht	Codon 824	Val > Val
CN-994	WT				Exon	C >T GTC>GTT ht	Codon 824	Val > Val
HD	WT				WT			

WT: wild type; IVS: intronic variation sequence; ht: heterozygous; hm: homozygous. CU = cervical cancer, CN = non-neoplastic cervix, HD = healthy donor lymphocytes. The polymorphisms were presented as ht = heterozygous or hm = homozygous. [RefSeq accession D50017]. All patients were wild type in exon 12, and 10 out of 17 had the silent mutation C >T in codon 824 GTC>GTT, Val>Val [rs10015469]. 2 samples of non-neoplastic cervical tissue presented the intronic variation C>A IVS12+22 [rs2307051] and all 3 presented the silent mutation in codon 824 [rs10015469]. In this table we show the results of only one lymphocytes donor, but all 8 donors were wild type for exon 12, and 3 out of 8 presented the polymorphism at codon 824.

### Cell proliferation

**Figure 3:**
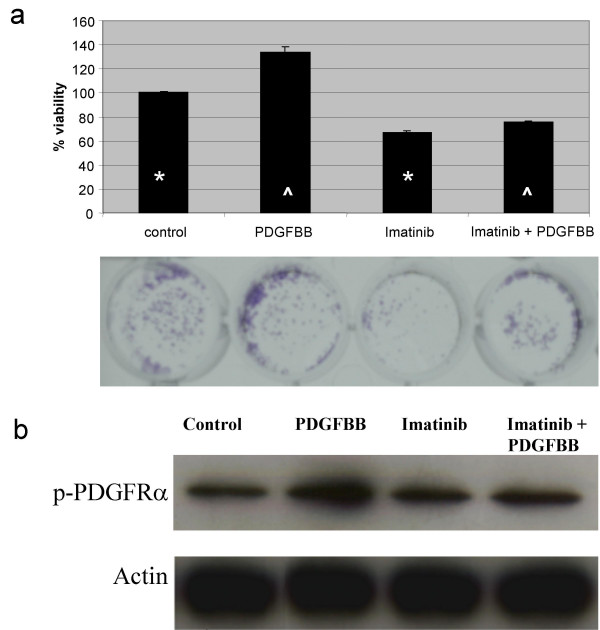
**a) **Effect of imatinib in cervical cell line growth. CaLo cells were grown for two days in the absence of serum and then re-plated for treatment with imatinib, PDGFββ or both for five days. PDGFBB stimulated cell gowth as compared to the untreated control. On the contrary imatinib induced growth inhibition and when cells were co-treated with the ligand and imatinib, the growth-inducing activity of the PDGFBB was partly blocked by imatinib (comparison are marked with * or **^**, all these differences were statistically significant p < 0.001). Their corresponding pictures of the plates stained with crystal violet is shown below. **b) **Inhibition of phosphorylation of PDGFRα by imatinib. CaLo cells were stimulated with PDGFBB at 10 ng/mL, for 10 min; treated with imatinib at 10 μM for 2 hours; treated with imatinib for two hours and then with PDGFBB for 15 min; or no treatment as control. Equal loading confirmed with actin. Receptor phosphorylation is increased in the PDGFBB lane and inhibited by imatinib treatment.

In order to investigate whether the PDGF system has a role in regulating cell growth, the cell line CaLo which co-express three family members, was grown for two days in the absence of serum and then re-plated for treatment with imatinib, recombinant PDGFBB or both for five days. The results show that the ligand BB led to a 37% growth stimulation as compared to the untreated control (p < 0.001). On the contrary imatinib induced a statisticaly significant growth inhibition as compared to untreated control (p < 0.001); and when cells were co-treated with the ligand and imatinib, the growth-inducing activity of the PDGFB was partly blocked by imatinib (p < 0.001), (Figure 3a).

### PDGFRα phosphorylation

The growth assays clearly demonstrated that in this model, cells are responsive to the growth stimulatory effect of the PDGFBB which can be partly blocked by the PDGFR kinase inhibitor imatinib. To assess whether this effect was associated with receptor activation, serum deprived CaLo cells were transiently stimulated with the PDGFBB for 15 min in the presence or absence of imatinib, and then analyzed by western blot with an antibody against the phosphorylated PDGFRα. Figure 3b shows that in untreated control cells, there was a basal level of receptor phosphorylation which increased after stimulation with the growth factor, but was inhibited to a similar degree of untreated cells by imatinib treatment.

## Discussion

Treatment results of locally advanced cervical cancer are suboptimal despite the recent success of first line therapy with concurrent chemoradiation [31,32]; or with and cisplatin plus topotecan for recurrent and metastatic [33]. Nevertheless, it is necessary to evaluate newer forms of therapy. Preclinical and clinical development of newer agents targeting molecular defects present in tumors [34] open the way for the identification of molecular targets in cervical cancer.

The availability of the tyrosin kinase inhibitor imatinib which not only targets bcr/abl but also c-kit and PDGFR has led to investigate the participation of these two receptors in several solid malignancies, particularly in small cell lung cancer and prostate carcinoma which over-express the c-kit receptor. However, early results using imatinib as a single agent have not been encouraging [35-37] which suggests that the sole presence of the putative target may not be sufficient to achieve tumor responses, besides, it is necessary to demonstrate that the pathway is functional and of relevance for one or more characteristics of the malignant phenotype in the tumor to be targeted.

So far, the role of the PDGFR pathway for the development of cervical cancer has been only studied in *in vitro *models, derived from the observation of the interaction between the E5 papilloma virus protein with the PDGFR [19,20]. There is a single study showing that the four primary cervical cancers analyzed by immunohistochemistry expressed the PDGFRβ [22]. In several tumors, where the participation of PDGF family has been demonstrated, a simultaneous expression of the receptors and the ligands seems to be important, suggesting an autocrine stimulation mechanism [38]. In the present study we evaluated a larger sample of primary tumors for the expression of the ligands and receptors of PDGF at both the malignant and stroma components of the biopsy. The expression of these growth factor family members in stroma is important as they are regulators of mesenchymal cell proliferation and migration during development, and therefore could play important roles in stromal fibroblast recruitment and tumor progression [39,40].

Our results indicate that around half of the tumors express the PDGFRα, PDGFRβ and PDGFA, in both tumor and stroma as shown in Figure 1. We found no expression of the B ligand in tumor samples, which was surprising as 5 out of 8 cervical cancer cell lines expressed this ligand. In addition, both ligands have been found expressed in a number of other tumor types [9]. Whether this feature is unique to primary cervical cancer tumors merits further study.

Interestingly 78% of the primary tumors express either the alpha or beta receptor. This elevated frequency of expression and co-expression highly suggest autocrine and paracrine functioning in cervical cancer tumors. This high frequency of expression and co-expression also occurred in the cervical cancer cell lines studied. It has been shown that the sole expression of PDGF receptors correlates with known adverse prognostic factors such as axillary lymph node metastases in breast cancer [28]. However, in this study we found no correlation between any combination of PDGF members expression with neither clinical characteristics of patients nor survival (data not shown). This observation does not exclude the possible prognostic significance of these receptors in cervical cancer. We also studied the mutational status of the *PDGFRα*, based on the identification of activating gene mutations within a subset of GISTs that lacked *KIT *gene mutation, and where constitutive phosphorylation of PDGFRα was observed, and the corresponding PDGFRα isoforms demonstrated ligand-independent kinase activity [29]. These observations have profound implications for the treatment of solid tumors with imatinib, as some of these mutations confer either sensitivity or resistance to this tyrosine kinase inhibitor [41]. This can be explained on the lack of differences in the activation of downstream signaling intermediates between *KIT*-mutant and *PDGFRα*-mutant tumors, suggesting that mutant *PDGFRα *provides oncogenic signals that parallel those of mutant *KIT *[29].

Our sequence results indicate that the cervical cancer cell lines and primary tumors analyzed showed a number of intronic and exonic variations, most of them previously unreported, (Tables 2 and 3). The mutation G>A in codon 571 leading to a Glu>Lys substitution found in three of the cell lines has not been reported; however, the deletion/substitution SPDGHE566-571R (already reported) involves codon 571 which corresponds to the juxtamembrane domain of the protein. The significance of this change in regard to imatinib sensitivity is unknown [42] but this deletion increases PDGFRα activation in the presence of PDGFA [29] suggesting that the mutation that we found, could affect the PDGFRα functioning. In an attempt to study the possible effect of this mutation on the protein, we used the simulation program PolyPhen [43] to predict whether this mutation, found in the cell lines, is likely to have biological meaning. The results indicate that the G>A at codon 571 of exon 12 is "bening". It is clear however, that this is only a theoretical approximation hence; the meaning of changes found must be investigated in experimental systems [44]. Besides, most of the intronic changes that we found are located in the proximity of the exon boundaries, and their possible meaning has to be studied. Regarding the samples from patients, we found the silent mutation at codon 824 already reported, in 10 out of 17 patients (59%). In the normal cervical samples, we found that 6 out of 11 presented the polymorphism at codon 824 (55%), indicating that this polymorphism is very common in our population. In exon 12, two samples presented the intronic change C>A IVS12+22 already reported [rs2307051] with no known consequence.

As stated above, the sole expression of the PDGFR or ligand is not an indication that the pathway is functional and that regulates the growth of the expressing tumor. Hence, we wanted to evaluate whether CaLo cell line which express the receptor alpha and both ligands is responsive to the growth factor and its inhibitor. Our results clearly demonstrate that while CaLo cells exhibit growth stimulation by PDGFBB, imatinib has on their own a small but significant growth inhibitory effect in serum-deprived cells, and that imatinib also partly blocks the PDGFB-induced growth stimulation. These effects on growth are likely due to activation of the PDGFR pathway as the results of western blots employing a primary antibody specific for the activated form of the receptor alpha indicate phosphorylation after the stimulation with PDGFBB, and that is back to unstimulated levels upon exposure to imatinib. It is noteworthy however, that in untreated cells despite being inhibited in their growth by imatinib, the basal level of phosphorylation of the receptor was unchanged after imatinib treatment, which may suggest that under the experimental conditions tested, the method has not enough sensitivity to detect a certain degree of phosphorylation inhibition. Even that we did not evaluate the activation of known downstream phosphorylation targets of the PDGFR pathway, the results demonstrate that as observed in other tumors such as osteosarcoma [45] and ovarian carcinoma [38], the blocking of this pathway could have therapeutical implications. Nevertheless, further functional characterization of the pathways either Src-PI3K-ERK, JNK1-p21 or other [46], known to be activated by PDGFR stimulation in other systems, is needed in order to optimize the potential use of PDGFR inhibitors in this malignancy. Likewise, is desirable to increase the number of primary tumors tested for activating mutations of the PDGFR to rule out the existence of activating mutations at this receptor that could be specifically blocked by PDGFR inhibitors. In addition, it remains to be explored whether the in *in vivo *blocking the receptor affects the interstitial pressure and would facilitate drug penetration into tumors.

## Conclusion

This study shows that members of the PDGF system are expressed in cervical cancer cell lines and primary cervical cancer tumors. In addition it is shown that the *PDGFRα *gene has several intronic and exonic changes but none of them biologically relevant. More interestingly, the results demonstrate that in a cervical cancer cell line, the system is functional as recombinant PDGFBB induces cell growth and receptor phosphorylation which can be blocked by imatinib. Despite the absence of activating mutations of PDGFRα, the co-expression of PDGF receptors and PDGFA in both the malignant cell component and stroma of the tumors, suggests the existence of an autocrine/paracrine growth stimulation loop which can be blocked by imatinib.

## Methods

### Cell lines and reagents

DMEM culture media and Fetal Calf Serum were purchased from Gibco BRL Life Technologies (Grand Island, New York). HeLa, CasKi, SiHa and C33A were obtained from the ATCC, ViBo, INBL, ViPa and CaLo carcinoma cell lines were kindly provided by Dr. Monroy (FES Zaragoza, UNAM, Mexico City). Cells were routinely grown in DMEM supplemented with 10% FCS at 37°C and 5% CO_2_. For immunocytochemistry analyses, cells were grown on two-chamber polysterene vessel Falcon^® ^(Becton Dickinson, NJ.) and subsequently formalin-fixed for 24 hrs at room temperature, then rehydrated in graded ethanol. Afterwards immunochemistry was performed as below described.

### Cervical cancer samples

Thirty-six paraffin-embedded tumor tissues from patients FIGO staged as IB2 to IIIB, treated with standard radiation concurrent with weekly cisplatin. Diagnosis was made on the basis of routine hematoxilin-eosin examination under light microscopy according to the World Health Organization criteria. Tumor specimens at diagnosis were taken before any treatment was instituted. For the mutational analysis of the PDGFRα 17 frozen samples of cervical cancer from an unrelated study were used. Biopsies from normal cervix and lymphocytes from 8 healthy donors were taken from a tissue bank.

### Immunohistochemistry

Immunohistochemistry for the PDGFRs and their ligands was performed using standard procedures. Briefly, sections were deparaffinized in xylene and rehydrated through graded ethanols to distilled water. The sections were immersed in Dako Epitope Retrieval Solution (10 mM citrate buffer, pH6) that had been preheated to 95°C in a water bath and then heat-treated at 95°C for 40 min. After a 20-minute cooldown period at room temperature, the sections were washed with PBS buffer, a procedure that followed every subsequent incubation. Endogenous peroxidase was blocked with Dako Blocking Buffer (0.3% hydrogen peroxide containing 15 mM sodium azide) for 5 min at room temperature. The sections were incubated for 30 min at room temperature with the following antibodies used at 1:75 dilution: PDGFA (monoclonal antibody sc-9974), PDGFB (polyclonal antibody sc-7878), PDGFRα (polyclonal antibody sc-338) and for PDGFRβ (monoclonal antibody sc-6252) all four from Santa Cruz Biotechnology. Bound primary antibody was labeled by incubating the slides with the Dako Visualization reagent (Dako EnVision + System, peroxidase (DAB, K-4007 and K4011). The sections were counterstained with hematoxylin and eosin. Tissues used as positive controls were placenta for both ligands, and normal skin for the receptors. In the negative controls, the primary antibody was ommited. Positivity was considered when 5% or more of the malignant cell population or the stroma stained. The analysis was performed by two pathologists.

### PCR-sequencing

For *PDGFRα *mutational analysis, genomic DNA derived from frozen samples and lymphocytes from 8 donors, was extracted following conventional techniques, with proteinase K digestion followed by phenol:chloroform extraction. *PDGFRα *DNA was amplified by PCR using the following primers: exon 12 forward: 5'-aagctctggtgcactgggactt-3' and reverse: 5'-attgtaaagttgtgtgcaaggga-3', product size 251 bp; exon 18 forward: 5'-tacagatggcttgatcctgagt-3' and reverse: 5'-agtgtgggaggatgagcctg-3', product size 212 bp. PCR was performed in 25-μL reactions containing 200 ng DNA, 10 mmol/L Tris-HCl (pH 8.3), 40 mmol/L KCl, 2 mmol/L MgCl_2_, 200 μmol/L of each dNTP, 1 μmol/L of each primer, and 0.25 U Taq polymerase (Applied Biosystems). PCR reaction was carried out on an 2400 Thermalcycler (Applied Biosystems). Initial denaturation at 94°C for 5 minutes was followed by 40 cycles of amplification and a final extension step (5 minutes at 72°C). The cycles included denaturation at 94°C for 30 seconds, annealing at 60°C for 30 seconds, and extension at 72°C for 30 seconds. PCR amplicons were purified using isopropanol precipitation and then sequenced in both forward and reverse directions from at least two independent amplification products. Purified DNA was diluted and cycle-sequenced using the ABI BigDye Terminator kit v3.1 (ABI, Foster City, CA) according to manufacturer's instructions. Sequencing reactions were electrophoresed on an ABI3100 genetic analyzer. Electropherograms were analyzed in both sense and antisense direction for the presence of mutations.

### Cell proliferation

CaLo cells were harvested in 75-cm^2 ^flasks, washed twice with PBS, and transferred in serum-free medium into 12-well plates at a seeding density of 1 × 10^3 ^cells/well. Each treatment group was plated in triplicate which consisted in: no treatment, recombinant human PDGF-BB at 10 ng/mL, Imatinib at 10 μM (freshly prepared in ddH_2_O) from a commercial presentation) and both, the ligand and imatinib. Fresh medium containing these treatments was replaced at day 3. Cells were harvested after five days of treatment and analyzed by the crystal violet method as reported [30]. Briefly, cells were incubated in 0.1% crystal violet for 30 min at room temperature, excess dye was removed by three brief rinses with ddH_2_O, the plates were air dried, and the dye was extracted with 10% acetic acid, which was then read in a plate reader (Wallac 1420; Perkin Elmer, Boston, MA, USA) at 570 nm. This method has shown to give an absorbance that correlates linearly with the number of cells over the range 1000 to 50,000 cells/well [30]. Assays were performed in triplicate and data are presented as a mean and SD. Statistical analysis of cell proliferation results was done using the ANOVA test.

### Western Blot analysis

CaLo cells were cultured with complete medium in 75-cm^2 ^flasks and when reached a 50–75% confluence, washed twice with PBS and replenished with serum-free medium for two days. Cells were then stimulated with PDGF BB at 10 ng/mL, for 10 min; treated with Imatinib at 10 μM for 2 hours; treated with imatinib for two hours and then with PDGF BB for 15 min; or no treatment as control. Cells were washed with ice-cold PBS solution and lysed in 0.2 mL of lysis buffer (Cell Signaling Technologies, Beverly, MA) at 4°C for 30 min. Lysates were cleared by a 10-min centrifugation at 10,000 × *g*, and protein determination was carried out according to the method of Bicinchoninic acid. Samples were subjected to 8% PAGE analysis after they were boiled for 5 min in sample buffer containing SDS. The separated proteins were transferred to an Hybond-P membrane (Amersham, Biosciences UK) and then blocked for 1 h in TBS (1× Tris-buffered saline) containing 5% nonfat milk. The membrane was then incubated with primary antibody in TBS overnight at 4°C. Primary antibody for p-PDGFRα, (Tyr 754 sc-12911-R, Santa Cruz Biotechnology, CA). Equal loading of protein samples (20 μg) was confirmed by incubating membranes with a primary antibody specific for actin (Santa Cruz Biotechnology). Membranes were washed and then incubated for 1 h at room temperature with secondary antibody. Bound antibody was detected using enhanced chemiluminescence reagent (ECL; Biosciences UK).

## Competing interests

The author(s) declare that they have no competing interests.

## Authors' contributions

LT-C performed the sequence analysis and contributed writing the manuscript; AC-B, AG-F and JC-V performed the inmunohistochemistry analysis; JM-T and A C-B did the western blot analyses; MC, contributed writing the manuscript; ER contributed with clinical data; and AD-G conceived and wrote the final version of the manuscript.
